# Home-based transcranial direct current stimulation in mild neurocognitive disorder due to possible Alzheimer’s disease. A randomised, single-blind, controlled-placebo study

**DOI:** 10.3389/fpsyg.2022.1071737

**Published:** 2023-01-03

**Authors:** Encarnacion Satorres, Joaquin Escudero Torrella, Elena Real, Alfonso Pitarque, Iraida Delhom, Juan C. Melendez

**Affiliations:** ^1^Faculty of Psychology, University of Valencia, Valencia, Spain; ^2^Neurology Department, Hospital General Universitario de Valencia, Valencia, Spain; ^3^Faculty of Psychology, Jaume I University, Castellón de La Plana, Spain

**Keywords:** ageing, memory, executive function, transcranial electric stimulation, neurocognitive disorder

## Abstract

**Introduction:**

Mild neurocognitive disorder (mNCD), a pre-dementia stage close to Mild Cognitive Impairment, shows a progressive and constant decline in the memory domain. Of the non-pharmacological therapeutic interventions that may help to decelerate the neurodegenerative progress, transcranial direct current stimulation (tDCS) shows beneficial effects on the learning curve, immediate recall, immediate verbal memory and executive functions. The purpose of this research was to study the effect of tDCS on general cognition, immediate and delayed memory and executive functions by comparing an active group with a placebo group of mNCD patients.

**Methods:**

Participants were 33 mNCD due to possible AD, randomly assigned to two groups: 17 active tDCS and 16 placebo tDCS. Ten sessions of tDCS were conducted over the left dorsolateral prefrontal cortex. Several neuropsychological scales were administered to assess the primary outcome measures of general cognitive function, immediate and delayed memory and learning ability, whereas the secondary outcome measures included executive function tests. All participants were evaluated at baseline and at the end of the intervention. Mixed ANOVAs were performed.

**Results:**

Significant effects were obtained on general cognitive function, immediate and delayed memory and learning ability, with increases in scores in the active tDCS group. However, there were no significant effects on executive function performance.

**Conclusion:**

The present study demonstrated the effectiveness of tDCS in an active tDCS group, compared to a placebo group, in improving general cognition and immediate and delayed memory, as previous studies found. Taken together, our data suggest that tDCS is a simple, painless, reproducible and easy technique that is useful for treating cognitive alterations found in neurodegenerative diseases.

## Introduction

Neurodegenerative diseases that cause impairments in cognitive functions are currently one of the main health problems in the older adult population (Mayeux and Stern, 2012), with Alzheimer’s disease (AD) being the most frequent cause (Rosenberg et al., 2020; McKeown et al., 2021). Memory loss is the most common initial symptom of AD, and although various therapeutic approaches have been proposed, none of them is sufficiently effective in slowing the progression of the disease (Lynch, 2020).

Mild neurocognitive disorder (mNCD; American Psychiatric Association, 2013), similar to the concept of mild cognitive impairment (MCI), is a pre-dementia stage (Sperling et al., 2011) that shows an objective progressive and constant decline in the memory domain, with no evidence of other aetiologies that could be responsible for it and without interfering with the capacity for independence in performing daily activities. Non-pharmacological therapeutic interventions during these stages may halt or at least slow down the neurodegenerative progress, thus preserving clinically unobtrusive stages as long as possible (Thams et al., 2020). Intervention techniques such as transcranial direct current stimulation (tDCS) may be a therapeutic option for maintaining cognitive functions that deteriorate throughout the course of the disease.

tDCS is based on the induction of low-intensity direct electric current (usually 1–2 mA) through electrodes placed on the scalp (Liu et al., 2017), and it is able to alter neuronal activity by crossing the calotte bone. Its main mechanism involves the modulation of the neuron’s resting potential, facilitating or inhibiting its depolarisation upon arrival of a stimulus (internal or external) and, thus, modifying the excitability of the cortex (Nitsche et al., 2008). Stimulation of the area underlying the anode (positive) reduces the resting potential and facilitates the discharge of the neuron upon arrival of a stimulus (facilitating effect), which can lead to an increase in brain activity. Cathodal stimulation (negative) is hyperpolarising and conditions an inhibition of the underlying area, with the neuron requiring the arrival of a stimulus of greater intensity in order to depolarise (Kalu et al., 2012).

Thus, tDCS changes the probability that an incoming action’s potential will generate postsynaptic firing, either in parallel to stimulation or a short time after it. Specifically, Prehn and Flöel (2015) proposed that, through this mechanism, memory traces are laid down in neuronal pathways. If a neuronal pathway is activated, a permanent change in this network would occur, thus allowing the information to be more easily retrieved or remembered. Di Lorenzo et al. (2018) noted that AD can be considered the result of a disconnection between different neuronal systems, suggesting a dysfunction of large-scale networks underlying memory processes. This suggestion leads to the idea that tDCS could have potentiating effects on neural circuits that act on various memory networks, favouring the functional restoration of these altered networks. Therefore, tDCS would be able to ameliorate cognitive dysfunction in MCI and temporarily reverse pathological brain activity (Meinzer et al., 2015).

tDCS induces effects that resemble the synaptic plasticity of glutamatergic connections (namely, synaptic long-term potentiation and long-term depression) (Nitsche et al., 2003), and studies have found that anodic tDCS strengthens the connections between remote, but functionally associated, brain areas (Polanía et al., 2011; Meinzer et al., 2012). Di Lorenzo et al. (2020) showed that, in patients with AD, there is a consistent long-term potentiation-like cortical plasticity alteration assessed with transcranial magnetic stimulation. This idea leads us to propose that tDCS could improve the synaptic plasticity of these circuits and, therefore, improve memory and learning. These effects are especially appropriate in patients who need to improve their ability to recall and make connections between memories, which is the case in patients with cognitive impairment. For this reason, much of the research to date has been directed to the temporal cortex, due to its important role in memory consolidation, and more specifically, to the dorsolateral prefrontal cortex (DLPFC), due to its important role in memory encoding, executive control functions and working memory. Some studies have shown that tDCS over the DLPFC is able to improve cognitive performance in both healthy and neuropsychiatric samples (Shin et al., 2015). As Tremblay et al. (2014) stated in their review, when using tDCS over the DLPFC with a specific set of parameters, it is possible to modulate a specific cognitive function such as memory performance and learning, working memory performance, word retrieval or executive function performance. Recently, Li et al. (2021), using transcranial magnetic stimulation over the DLPFC in AD patients, found a significant improvement in cognitive skills that correlated with improved cortical plasticity.

tDCS has a very favourable tolerability and safety profile. The most frequently reported side effects are a tingling or itching sensation under the electrodes, headache or tiredness (Fertonani et al., 2015). However, the slight itching sensation at the beginning of stimulation usually disappears during the process (Ambrus et al., 2012), and the patient does not usually notice anything. It is safe to say that no structural damage to the underlying brain tissue occurs when following current stimulation protocols (Nitsche and Paulus, 2001).

With regard to its application in older adults with and without neurodegenerative pathologies, evidence supporting the efficacy of tDCS for cognitive functions such as memory and executive functions is inconsistent.

Anodic stimulation of the temporo-parietal area in healthy older adults shows positive effects on the learning curve and immediate recall (Flöel et al., 2012), and these effects are maintained up to 1 week later, suggesting that the technique facilitates more efficient encoding and minimises the deterioration of learned information. It has been proposed that the memory improvements observed may be related to effects on the acquisition and/or consolidation of memory traces through increased plasticity (Coffman et al., 2014). Anodal stimulation in MCI patients also shows improved performance on immediate verbal memory (Fileccia et al., 2019). Several meta-analyses (Wang et al., 2021; Majdi et al., 2022) have confirmed the efficacy of anodal stimulation on the DLPFC in MCI and AD patients on memory tests. However, a recent review (Ciullo et al., 2021) reported that evidence of a positive effect on memory is inconclusive in AD and weak in MCI.

In the case of the executive functions, anodic stimulation on the left DLPFC has been found to enhance the effectiveness of working memory training (Mancuso et al., 2016). Combining working memory training with cognitive stimulation strengthens the gains, compared to the exclusive administration of stimulation (Nissim et al., 2019). Meta-analyses have pointed to different results depending on the population to which the technique has been administered. In healthy older adults, significant but small effects of anodal stimulation on the left DLPFC have been reported (Mancuso et al., 2016; Lee et al., 2021). In MCI patients, slightly significant benefits of anodic stimulation have been found for executive functions, pointing to its potential clinical application in these patients (Xu et al., 2019).

Due to the lack of consensus and the contradictions in the literature, it seems necessary to try to obtain information about the efficacy of anodic tDCS. Given the high prevalence of mNCD and the inexistence of effective treatments, anodic tDCS would provide important social and economic benefits (Fileccia et al., 2019), mainly due to its easy administration, safety and low cost. The present study aimed to examine whether active tDCS has an effect on general cognition, immediate and delayed memory tasks and executive functions. Specifically, we hypothesised that active stimulation, compared to placebo stimulation, would improve performance in the cognitive domains assessed in patients with mNCD due to possible AD.

## Materials and methods

### Study design and participants

We carried out a randomised, single-blind and controlled-placebo study consisting of 10 sessions of active anodal tDCS on the left DLPFC, compared to a sham or placebo group. Participants with mild neurocognitive disorder due to possible Alzheimer’s disease were assessed before and after the intervention. At the end of the first assessment, inclusion and exclusion criteria were checked, and patients were randomly assigned to the active tDCS group or the sham group.

Before randomisation, participants who were eligible for the study had to meet the following criteria:

1. Age > 65 years.
2. A score equal to 3 on the Global Deterioration Scale (GDS; Reisberg et al., 1982).
3. A score of more than 18 and less than 26 on the Mini-Mental State Examination (MMSE; Folstein et al., 1975).
4. Presence of mild neurocognitive disorder due to possible Alzheimer’s disease, according to DSM-5 criteria (American Psychiatric Association, 2013). For this diagnosis, there had to be: A. Evidence of a moderate cognitive decline based on: 1. concern about cognition expressed by the individual or an informant who knows him/her; 2. lower performance than expected on an objective assessment; B. Cognitive deficits must not interfere with the ability to be independent in activities of daily living; C. They must not occur exclusively in the context of delirium and D. They would not be better explained by another mental disorder. In addition, for a diagnosis of possible Alzheimer’s disease, evidence of a genetic mutation causing Alzheimer’s disease is not required, but the following three criteria must be met: a. clear evidence of a decline in memory and learning, b. a progressive, gradual, and steady decline in cognitive ability without prolonged plateaus, and c. no evidence of another causal aetiology.

If one or more of the following criteria were present in the randomisation, potential participants were excluded: a score of 4 or more on the GDS and/or a score of less than 18 on the MMSE. In addition, subjects with contraindications to tDCS (intracranial metallic implants, intracranial hypertension), significant cerebrovascular disease or severe psychiatric symptoms were excluded.

Participants were recruited from the Neurological department of the Consorcio Hospital General Universitario of Valencia. Initially, 38 patients were contacted. Three were excluded because they had an MMSE of less than 18, and two patients decided not to participate after being assessed the first time. The final sample consisted of 33 subjects, 17 men and 16 women (51.5 and 48.5% respectively), between 65 and 88 years old (*M* = 75; *SD* = 6.08). All of them signed the informed consent form at the beginning of the study. This study was approved by the Human Research Ethics Committee of the University of Valencia.

**Figure 1:**
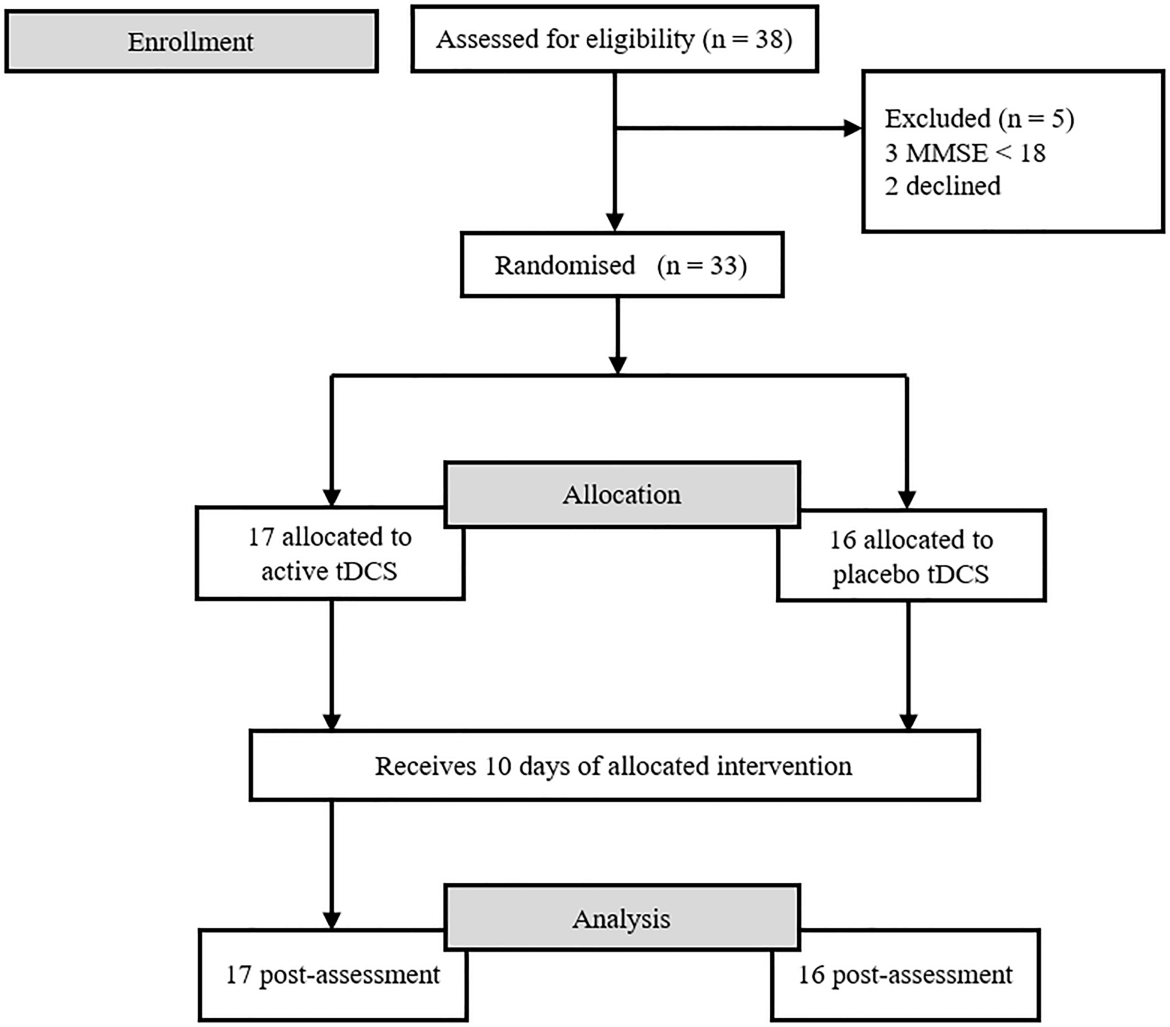
Flow diagram of trial profile.

The eligible patients were allocated to the anodal and sham tDCS groups through stratified block randomisation. Using a random number system, participants were randomly assigned to the experimental groups (anodal vs. sham) at a 1:1 ratio, with gender as the stratum. Participants did not know to which group they had been assigned until the intervention ended, given that this was a single-blind study. A flowchart of the study is shown in Figure 1.

The active anodal tDCS group consisted of 17 participants, and the sham or placebo group consisted of 16 participants. Table 1 presents the main data and comparison tests for the two groups, which were matched on their characteristics.

**Table 1:** Means (and *SD*) of demographic indices comparing active and sham groups

	**ACTIVE** **(n = 17)**	**SHAM** **(n = 16)**	**Significance** **(*p-value*)**
Age	76.6 (5.7)	73.4 (6.2)	0.139
Gender	9 men/8 women	8 men/8 women	0.862
Education (years)	10.35 (3.9)	10.8 (4.6)	0.769
MMSE	23.88 (3.2)	22.94 (3.9)	0.402

### Measures

The primary outcome measures were general cognitive function, immediate and delayed memory and learning ability. The secondary outcome measures included executive function tests. The selection of primary outcomes was based on the DSM-5 criterion (American Psychiatric Association, 2013) that states that mild neurocognitive disorder due to possible Alzheimer’s disease should show clear evidence of a decline in memory and learning. The assessment of memory and learning is a main criterion in the characterisation of the pathology, and we aimed to analyse the possible change on measures of these variables. In addition, given that the progression of these patients to major neurocognitive disorder due to Alzheimer’s disease is usually accompanied by a deterioration in executive control, changes in the measures that assess this variable were proposed as a secondary outcome.

The Mini-Mental State Examination (MMSE; Folstein et al., 1975) was used as an index of global cognitive functioning; the maximum score is 30 points. This test was designed to estimate the existence and severity of cognitive impairment; it is a brief, quantitative test that measures general cognitive function.

Test de Aprendizaje Verbal Complutense (TAVEC; Benedet and Alejandre, 1998). On this test, the evaluator reads a list of 16 words that the participant has to repeat. The list is repeated five times (trials), and after 20 min, the participant is again asked to remember the 16 words. The test was administered in order to evaluate the participant’s immediate memory (Trial 1), learning ability (analysing Trial 5 and the total number of correct answers on the five trials) and delayed memory.

Memory Alteration Test (M@T; Rami et al., 2007). The M@T provides efficient and valid screening for A-MCI and early-stage AD. The test evaluates different abilities such as encoding, orientation, semantic memory and free recall. The maximum score is 50 points.

Direct and inverse digits of the Wechsler Intelligence Scale for Adults-III (Wechsler, 2001). These tests assess attentional capacity by exposing the participant to increasing amounts of information. On the direct digits task, which is used to assess immediate recall, the subject must repeat the sequence of numbers in the same order in which they are read by the examiner. On the inverse digits task, which assesses working memory and mental flexibility, the subject must say the digits backwards from the way they were presented by the examiner. Both tests are evaluated in the same way, assigning one point for each correct item, with a maximum score of 16 on both tests.

Rey’s Complex Figure (Rey, 1984). This test is used to evaluate both memory and executive capacity, such as planning, motor skills, working memory and visuoconstructive and spatial ability. The subject has to attentively reproduce a complex geometric drawing and later reproduce it after a 3-min delay.

Semantic fluency and phonological fluency subtests of the Barcelona Test Revised (TBR; Peña-Casanova, 2005). The semantic fluency subtest requires the subject to evoke the maximum number of words linked to a specific category, “animals,” in 1 min. In the case of phonological verbal fluency, the subject is asked to recall the maximum number of words beginning with the letter “p” 3 min or less. This test evaluates the ability to access and recall elements from the lexical and semantic store. Among the processes involved, information processing speed, cognitive flexibility and working memory have been pointed out.

### Intervention

Stimulation was performed using an HDC stimulator (Newronika TM, Milan, Italy), which is battery-driven and delivers a direct current. The current intensity was 2 mA, and the stimulation lasted 20 min. In addition, a current ramp was delivered for 30 s prior to the start and end of stimulation. A pair of 25 cm^2^ rubber electrodes transferred the direct current. These electrodes were inserted into sponge pads soaked with sterile water. The electrodes and sponges were placed in a neoprene headcap with predefined and clearly annotated positions, based on a subset of the international 10–10 EEG system. The anode was placed on F3 at the left dorsolateral prefrontal cortex (DLPFC), and the cathode was placed on the right frontal lobe (Fp2). Electrode placement and session duration were identical in the active and placebo tDCS conditions. However, in the placebo tDCS condition, at the end of the onset ramp, the current was automatically turned off for 20 min and then turned on again for the last 30 s.

### Procedure

The Neurology Department recruited patients who reported memory complaints and could be candidates for the study and briefly informed them of the study’s objective. After initial telephone contact, an appointment was made to explain the research procedure. After providing their informed consent, the patients participated in a demographic interview to check the eligibility criteria. Performance in the cognitive domains was then assessed; participants had to obtain test scores within the range of 1–2 standard deviations (SD) below the mean to be included. A multidisciplinary team (neurologists and psychologists) performed the diagnosis and made clinical decisions. Participants who met the eligibility criteria were randomly assigned to the anodal tDCS and sham groups. After assigning patients to the groups, an appointment was made for the home-based intervention, which lasted 10 consecutive days. Two technicians (psychologists) who were specifically trained in the technique performed and monitored the home intervention daily in the patients’ homes. In addition, to check the stimulations carried out, the HDCstim^®^ stimulator was connected to the HDCprog, which has a “treatment report” in its menu. These reports show: (a) the time and date of each stimulation, (b) the average impedance of each stimulation a suitable impedance range would be from 4 to 12 kOhm and (c) the result of the treatment (completed, failed or cancelled). On the last day, after the stimulation ended, the evaluation was performed again.

### Data analysis

An *a priori* power calculation for a repeated-measures ANOVA within-between interaction called for a total sample of 32 participants in order to detect an effect size of 0.30 with 90% power, with alpha set at 0.05. Power calculations were carried out using GPower 3.1.7.

To analyse the sociodemographic variables, we used independent sample *t*-tests and chi-square tests. To analyse the cognitive variables, mixed ANOVAs with 2 groups (active vs. sham; between-subjects) × 2 sessions (before vs. after intervention; within-subjects) were conducted. *Post-hoc* simple effects tests were conducted to analyse the significant interactions. Because we performed the same ANOVAs on the primary and secondary dependent variables, we applied the Bonferroni correction to control the type I error. Data were analysed using SPSS 28.

## Results

Table 2 shows the main and interaction effects of the mixed ANOVAs with 2 groups (active vs. sham; between-subjects) × 2 sessions (before vs. after intervention; within-subjects). Given that the effectiveness of the tDCS should be observed by finding a significant interaction between the two independent variables (where the active group would improve throughout the sessions, whereas the sham or control group would not), for the sake of simplicity, only the significant interactions are analysed below. The means (and SD) of the group (active vs. sham) × session (before vs. after intervention) interactions are shown in Table 3.

**Table 2:** Main and interaction effects of the mixed ANOVAs with two groups (active vs. sham; between-subjects) × 2 sessions (before vs. after intervention; within-subjects).

	Main effect sessions			Main effect group			Interaction sessions × Group		
	*F*(1, 31)	*p*	η^2^	*F*(1, 31)	*p*	*η* ^2^	*F*(1, 31)	*p*	*η* ^2^
MMSE	4.38	00.045	00.124	22.97	00.097	0.086	9.89	0.004	0.242
TAVEC trial 1	0.12	00.724	00.004	0.78	00.383	0.025	23.54	<0.001	0.432
TAVEC trial 5	5.33	00.028	00.147	7.17	00.12	0.188	11.5	0.002	0.271
TAVEC total	6.97	00.013	00.184	6.34	00.017	0.170	6.34	0.017	0.170
TAVEC delayed	10.12	00.003	00.246	3.97	00.055	0.114	8.34	0.007	0.212
M@T	9.85	00.004	00.241	6.06	00.020	0.164	17.63	<0.001	0.363
Direct digits	4.67	00.039	00.131	2.27	00.141	0.068	8.15	0.008	0.208
Inverse digits	0.1	00.750	00.003	2.21	00.147	0.067	0.57	0.455	0.018
Copy Rey’s Figure	0.54	00.467	00.017	1.15	00.291	0.010	2.39	0.132	0.072
Delayed Rey’s Figure	11.44	00.002	00.270	0.01	00.994	0.001	0.1	0.972	0.001
TBR semantic fluency	0.42	00.524	00.013	1.32	00.258	0.041	6.48	0.016	0.173
TBR phonological fluency	1.26	00.269	00.039	0.34	00.564	0.011	0.24	0.624	0.008

**Table 3:** Means (and SD) of the dependent variables as a function of groups (active vs. sham) and sessions (before vs. after intervention).

	Active		Sham (Control)	
	Before	After	Before	After
MMSE*	23.88 (3.24)	26.06 (3.27)	22.94 (3.91)	22.50 (5.19)
TAVEC trial 1*	3.24 (1.56)	4.18 (1.91)	3.62 (1.59)	2.81 (1.56)
TAVEC trial 5*	6.06 (2.79)	7.71 (2.29)	4.94 (2.02)	4.63 (2.42)
TAVEC total	25.00 (8.86)	30.29 (10.94)	22.31 (7.14)	22.44 (8.66)
TAVEC delayed*	2.41 (3.14)	3.71 (3.67)	1.19 (1.68)	1.25 (1.69)
M@T test*	27.94 (6.29)	34.00 (8.48)	23.88 (9.67)	23.00 (11.37)
Direct digits*	7.12 (1.45)	8.47 (2.07)	6.87 (2.73)	6.69 (1.89)
Inverse digits	4.59 (3.10)	4.76 (1.95)	3.81 (2.14)	3.38 (2.19)
Copy Rey’s Complex Figure	41.65 (41.57)	52.29 (40.50)	62.69 (35.83)	58.91 (38.86)
Delayed Rey’s Complex Figure	5.71 (5.30)	8.71 (6.89)	5.65 (6.86)	8.72 (10.04)
TBR semantic fluency	13.53 (4.05)	14.00 (8.52)	17.41 (9.93)	11.69 (5.71)
TBR phonological fluency	20.82 (12.57)	22.59 (12.47)	18.56 (15.44)	19.25 (15.90)

Asterisks indicate significant interaction based on Bonferroni correction.

### General cognition

MMSE *post-hoc* simple effects tests to analyse the significant interaction (Table 3) showed that the groups did not differ significantly before the intervention (*p* = 0.45), but after the intervention, the active group scored significantly higher than the placebo group (*p* = 0.02). Moreover, the sham group did not improve across the sessions (*p* = 0.47), but the active group did (*p* = 0.001).

### Memory

Because we conducted six similar ANOVAs on the six memory variables, only effects with *p* ≤ 0.008 (Bonferroni correction) were considered significant.

*Post-hoc* simple effects tests to analyse the significant interaction in TAVEC 1 trial 1 (Table 3) showed that the groups scored similarly before the intervention (*p* = 0.48), but after the intervention, the active group scored marginally higher than the placebo group (*p* = 0.03). In addition, the control group showed a significant decrease across sessions (*p* = 0.004), whereas the active group showed a significant increase (*p* = 0.001).

Moreover, when analysing the significant interaction in TAVEC trial 5 (Table 3), *post-hoc* simple effects tests showed that the groups scored similarly before the intervention (*p* = 0.20); however, the active group’s score after the intervention was significantly higher than the sham group’s score (*p* = 0.001). The sham group did not improve their scores across sessions (*p* = 0.46), but the active group did (*p* < 0.001).

With regard to the total number of correct answers on the five TAVEC trials, although the means were in the expected direction (Table 3), the interaction was marginally significant (*p* = 0.017), probably due to the small sample size.

For the last TAVEC subtest (delay), the results obtained on the *post-hoc* tests indicated that the scores of the groups before the intervention were similar and showed no significant differences (*p* = 0.18); after the intervention, the active group’s score was marginally higher than the sham group’s score (*p* = 0.021). The sham group did not improve significantly across the sessions (*p* = 0.84), whereas the active group did (*p* < 0.001).

Regarding the analysis of the M@T test, *post-hoc* simple effects tests showed (Table 3) that the groups scored similarly before the intervention (*p* = 0.16), but after the intervention, the active group scored significantly higher than the sham group (*p* = 0.003). Moreover, the sham group did not improve across the sessions (*p* = 0.47), but the active group did (*p* < 0.001).

For the last primary outcome, the analysis of direct digits (Wechsler test), *post-hoc* tests revealed (Table 3) that the groups scored similarly before the intervention (*p* = 0.75), but after the intervention, the active group scored marginally higher than the simulated group (*p* = 0.015). In addition, the sham group did not improve over the sessions (*p* = 0.63), but the active group did (*p* = 0.001).

Overall, these results demonstrate the efficacy of the tDCS in improving the patients’ memory.

### Executive functions

Because we conducted six similar ANOVAs on the six secondary variables, only effects with *p* ≤ 0.008 (Bonferroni correction) were considered significant.

In the case of these secondary variables, none of the interactions reached statistical significance, which would indicate that tDCS was ineffective in improving the executive functions.

### Safety and tolerability

All the participants tolerated stimulation, and apart from a brief tingling sensation under the electrodes, no adverse events were reported during stimulation.

## Discussion

The aim of the present study was to compare the effects of an active anodal tDCS intervention on general cognition, immediate and delayed memory and executive functions in patients with mild NCD due to possible AD. tDCS was found to be effective in improving general cognition and immediate and delayed memory. However, no significant effects on performance on executive function tests were observed.

The results obtained for general cognition are consistent with previous research that applied anodal stimulation on the left DLPFC (Khedr et al., 2014; Im et al., 2019). With a protocol similar to the one used in this research, it can be argued that the improvement mechanism is probably multifactorial; the technique may facilitate the activation of the cognitive reserve that is still present in patients at the onset of cognitive impairment (Khedr et al., 2014). A more recent study administered stimulation to patients with mild AD for 6 months and concluded that this technique can prevent the worsening of global cognitive functioning in patients who begin to show symptoms of significant impairment (Im et al., 2019). Several meta-analyses have reported the efficacy of tDCS in improving overall cognition in patients with AD (Hsu et al., 2015; Majdi et al., 2022), patients with mild to moderate AD (Chen et al., 2022) and patients with MCI, with anodal stimulation also being the most effective method (Saxena and Pal, 2021). However, other meta-analyses (Inagawa et al., 2019; Gu et al., 2022) of patients with MCI and AD reported no efficacy. These contradictory results may be due to a lack of consensus in the methodology and small sample sizes (Chang et al., 2018).

In the memory domain, the present study demonstrated the effectiveness of tDCS compared to a placebo. These results converge with previous studies that obtained significant improvements in the active group on memory recall and immediate and delayed recall in patients with MCI after stimulation on the left DLPFC (Murugaraja et al., 2017; Gomes et al., 2019). Other research (Yun et al., 2016; Fileccia et al., 2019) reported that when anodic stimulation was applied on the DLPFC in subjects with MCI, memory performance increased. In this regard, some authors have concluded that tDCS could have the potential to restore brain activity in patients at risk of developing dementia (Ciullo et al., 2021). However, other research (Cruz Gonzalez et al., 2018) did not observe significant improvements in short-term memory performance.

MCI patients have impaired functional connectivity between the DLPFC and several cortical and subcortical regions involved, and this would affect various cognitive functions, including learning, which is closely related to memory (Liang et al., 2011). In AD, it has been hypothesised that the DLPFC is a compensatory brain resource that supports memory function, and that this compensatory mechanism is strongest in the prodromal and mild stages of the disease, decreasing with the progression to more advanced stages of AD (Gigi et al., 2010). Thus, as suggested in several neuroimaging studies, the left DLPFC should be a major area for the stimulation of declarative memory (Cabeza and Nyberg, 2000). Furthermore, the DLPFC has been found to be involved in the encoding, manipulation, organisation and retrieval of verbal content (Cabeza et al., 2002; Nyberg et al., 2003; Barbey et al., 2013), and these results have been confirmed in review studies (Manenti et al., 2012).

In contrast to these cognitive domains, no significant effects were obtained on the executive function tests. This result is similar to those obtained in other studies (Khedr et al., 2014; Fileccia et al., 2019; Gomes et al., 2019) that did not observe significant increases in executive function performance after anodic stimulation on the DLPFC. A meta-analysis (Chen et al., 2022) revealed that stimulation of temporal-lobe-related brain regions produced better cognitive improvement than left DLPFC stimulation, and it concluded that AD patients may benefit more than MCI because tDCS treatment did not provide significant benefits in the latter. Whereas with disease progression AD patients may also experience impairments in executive function, MCI is considered an initial stage of Alzheimer’s disease in which memory shows impairment while executive function may be preserved. This argument could be the key to explaining why, in this study, executive function performance did not improve after stimulation, and it is plausible that no significant effects on performance would be observed if performance was already adequate at baseline. Huo et al. (2018) were unable to find statistical evidence for an effect of anodal stimulation on the executive function of healthy older adults. This result may be due to the absence of simultaneous cognitive training, and it suggests that, at least in the executive function domain, tDCS may be an adjuvant to cognitive training rather than a cognitive modulator with an independent effect. The cognitive enhancement potential of tDCS may occur by boosting the efficiency of training. However, other studies have found effects of the technique. Applying tDCS over the DLPFC for 6 months in AD produced stability in the scores of the active group, whereas these scores decreased in the sham group (Im et al., 2019). In another previous study, anodic stimulation of the DLPFC combined with cognitive stimulation was administered in patients with MCI, obtaining better executive function performance (Cruz Gonzalez et al., 2018). Furthermore, the DLPFC is specialised in executive function (Otero and Barker, 2014) and hypo-activated in mNCD (Peelle et al., 2014), and so its stimulation could be essential in reducing hypoactivation.

In conclusion, given its easy home administration, tolerability, safety and low cost, tDCS could become a real treatment option for the maintenance of cognitive function in patients with mNCD [35]. However, evidence of its efficacy remains controversial (Ciullo et al., 2021). The high inter-individual variability in the effects of the technique (Button et al., 2013) and the lack of standardised parameters and measurements of current intensity, stimulation time, electrode placement and the number and frequency of sessions (Gomes et al., 2019) are some of the elements that hinder the generalisability of the results. Thus, further information is needed to help to consolidate the technique and its specific parameters and indicate which specific cognitive abilities might obtain a real and significant effect (Majdi et al., 2022) and which patients would benefit the most from the technique.

Finally, it should be noted that several clinical trials reinforce the idea that efficacy increases with multiple sessions and that the effects are believed to be cumulative (Ferrucci et al., 2014; Meesen et al., 2014). However, multiple sessions require subjects to travel to the clinic for each treatment, placing a significant and often insurmountable burden on patients and their caregivers and requiring significant provider time and cost, especially as the sample size increases (Charvet et al., 2015). These difficulties lead to high dropout rates (Valiengo et al., 2013).

Providing tDCS treatment at home can decrease the burden on patients and their families by eliminating the need to travel to medical or research facilities for each treatment session, thus promoting treatment adherence and compliance (Charvet et al., 2020). However, some research points out that the lack of remote supervision (optional telephone backup) seems to be a main factor in dropouts when tDCS is applied by patients or caregivers at home. Therefore, applying procedures such as remotely supervised, regular or even daily visits, as in this research, seems to be necessary, not only to assess clinical efficacy, but also to control the correct performance of the stimulations and prevent dropout (Palm et al., 2018).

### Limitations

Some limitations should be considered. Regarding to the sample, it would be interesting to increase the number of patients in order to improve the statistical power. In addition, it should be noted that, although the criterion of independence in instrumental activities differentiates MCI from AD patients, at a cognitive level, the differentiation between initial dementias is not always clear. Moreover, in relation to the design, this study was single-blind with pre- and post-intervention measures. It would have been advisable to apply a double-blind design to avoid possible researcher bias and obtain follow-up measures to find out whether the results are maintained, for example, 1 month after the intervention. In addition, the importance of including biomarkers in the diagnosis of the early stages of AD, which this study was unable to do, should be noted. Dubois et al. (2014) point out that the research criteria for the early stages of AD require the presence of at least one biomarker of Alzheimer’s pathology for identification (decreased Aβ1–42 along with increased T-tau or P-tau in CSF or increased tracer retention on amyloid PET). These authors consider pathophysiological markers to be indicators of Alzheimer’s pathology in the brain, rather than markers linked to disease stages. That is, they describe the presence of disease pathology at any stage and simplify the diagnostic approach by designating a single *in vivo* pathophysiological signature of AD, measured in CSF or using amyloid PET.

As future lines of work, the intervention could be administered to patients with major NCD in order to determine whether stimulation is also effective when there is significant cognitive impairment. In addition, it would be interesting to find out whether the changes at the cognitive level have a positive long-term effect on the quality of life and emotional level of the participants in the active group.

## Conclusion

This randomised, placebo-controlled study revealed significant results for general cognition and memory in patients with mNCD due to possible AD. This trial showed high safety and tolerability of tDCS, a simple, painless, reproducible, easy to administer and inexpensive technique. For all these reasons, its potential usefulness for treating the cognitive alterations in neurodegenerative diseases presents researchers with a great opportunity.

## Data availability statement

The raw data supporting the conclusions of this article will be made available by the authors, without undue reservation.

## Ethics statement

The studies involving human participants were reviewed and approved by the Human Research Ethics Committee of the University of Valencia H1526539449220. The patients/participants provided their written informed consent to participate in this study.

## Author contributions

JM and AP designed and drafted the work. AP and ID were in charge of carrying out the statistical analyses and writing the results. ES and JM contrasted the results obtained with other studies and wrote the discussion. JE, ES, and ER participated in the selection of the sample and the design of the stimulation based on tDCS. All authors carefully read the manuscript and fully approved it.

## Funding

This work was supported by Ministerio de Ciencia e Innovación (MCIN)/Agencia Estatal de Investigación (AEI)/10.13039/501100011033 (Grant PID2019-103956RB-I00) and Conselleria de Innovación, Universidades, Ciencia y Sociedad Digital of Generalitat Valenciana (Spain) (Grant GV/2021/174).

## Conflict of interest

The authors declare that the research was conducted in the absence of any commercial or financial relationships that could be construed as a potential conflict of interest.

## Publisher’s note

All claims expressed in this article are solely those of the authors and do not necessarily represent those of their affiliated organizations, or those of the publisher, the editors and the reviewers. Any product that may be evaluated in this article, or claim that may be made by its manufacturer, is not guaranteed or endorsed by the publisher.
